# Urinary Incontinence: Its Assessment and Relationship to Depression among Community-Dwelling Multiethnic Older Women

**DOI:** 10.1155/2014/708564

**Published:** 2014-03-25

**Authors:** Luciana Laganà, David William Bloom, Andrew Ainsworth

**Affiliations:** Department of Psychology, California State University, 18111 Nordhoff Street, Northridge, CA 91330, USA

## Abstract

Urinary Incontinence (UI) affects many older adults. Some of its deleterious consequences include stress, major depression, diminished quality of life, sexual dysfunction, and familial discord. Of the various mental health problems identified in the literature as being comorbid with UI, the most notable one continues to be depression. Despite a wealth of research contributions on this topic, the available literature is underrepresentative of ethnic minority older women. Culture has been shown to have a significant impact on a woman's perception of her own UI symptoms; this demonstrates the necessity for the recruitment of ethnically and culturally diverse samples when studying UI. In the present study, we determined the prevalence of UI among 140 community-dwelling, ethnically diverse older women (28.2%), discovered that our new UI screener is reliable, and did not find the UI-depression link to be significant. The clinical and research implications of our findings are discussed.

## 1. Introduction

Urinary Incontinence (UI) is a pelvic floor disorder leading to an involuntary loss of urine that commonly affects older adults [1]. Of the 23.7 percent of women in the United States of America (USA) living with a pelvic floor disorder, 15.7 percent of cases are accounted for by UI, with prevalence rates among older women between 23 and 31.7 percent [2]. With some estimates of undiagnosed UI in women placing prevalence rates as high as 50 percent, the economic ramifications of this condition are vast, with direct cost estimates in the USA exceeding $12 billion dollars [3]. Along with the monetary ramifications of UI, there are a number of psychosocial consequences associated with this disorder affecting both sufferers of UI and their caregivers. Some of the deleterious syndemic and comorbid consequences of UI include sexual dysfunction, stress, major depression, diminished quality of life, and familial discord [4–7]. With USA census population projections estimating that one in every five women will be over the age of 65 by 2020 [8], more research on health concerns prevalent within this population is needed in order for health professionals to better assess the needs and resources required to successfully accommodate, care, and treat older women.

### 1.1. Purpose and Rationale of the Present Study

In this study, we tackled the issue of researching older women's UI in three ways: first, for aim (1), given the major inconsistencies regarding prevalence of UI among nonclinical populations, we intended to estimate the prevalence of UI among a sample of nonclinical, community-dwelling women, all of whom are 60 years of age or older (*N* = 140). This sample is highly representative of the Los Angeles-based community from which the research participants were recruited. Second, we wanted to evaluate the feasibility of using a novel screening measure devised by the first author to rapidly and easily screen older women for UI symptoms, thus allowing clinicians to better assess the needs of their clients. Finally, we planned on testing our hypothesis regarding the comorbidity of depressive symptomatology and UI. Concerning this study's rationale, the rapid assessment of UI in older women is vital, given the range of underlying medical conditions associated with it. Although UI is not usually fatal, a missed diagnosis could prevent early detection of serious central nervous system and nephrological disorders [9]. Clinical populations may be assessed by 24-hour monitoring and pad testing, yet these methods are not always practical with nonclinical community-dwelling older women [10].

Explorations of depressive symptomatology among older women living with UI are critical to the promotion of accurate perceptions regarding the health and functioning of this population. Furthermore, such investigations along with others pertaining to UI must not overlook racial and ethnic factors [11]. We conducted a thorough review of the literature regarding UI (briefly summarized below) that has revealed a glaring neglect of non-European-American populations in research concerning the prevalence of UI and related psychosocial factors. Although evidence exists to suggest that cultural beliefs can affect how older women perceive UI, the development of UI-related instruments and interventions utilizing multiethnic community-dwelling populations has been greatly lacking [12–14]. Homogeneous samples pose external validity problems for most research [15], yet there is evidence suggesting that such issues are of immense concern with regard to UI where culture can have a significant impact on a woman's perception of her own urinary symptoms [16]. Thus, the creation of instruments and assessment techniques for UI that have been developed through the use of ethnically diverse sample populations is necessary to ensure proper UI diagnosis and competent care.

### 1.2. Literature Review

#### 1.2.1. Prevalence of UI

Precise prevalence estimates of UI across various populations are necessary if we are to employ effective UI treatment and management strategies [17]. Accurately assessing prevalence of UI among nonclinical samples has been especially difficult due to various sociocultural barriers to UI self-reporting. Some of the most common barriers to reporting in the literature are shame, embarrassment, the belief that UI is not a legitimate medical concern, and perceptions regarding aging and childbirth [3, 18, 19]. Studies conducted on attitudes towards UI have revealed the existence of a sharp contrast between the perceptions of laypersons and of the researchers who have published literature on UI [20]. Their findings show that older adults and their caregivers typically view UI as normal, irreversible, and indicative of incompetence, although the literature concerning UI identifies it as reversible and requiring treatment. The ramifications of such discrepancies are a profound association between UI and culturally undesirable traits, as Mitteness [20] eloquently stated: “Incontinence is a cultural symbol for the increasing dependencies of old age, dependencies that are much feared and resented in USA society, where tremendous emphasis is placed on independence even into advanced old age” (p. 188).

Unfortunately for the incontinent person, the above-mentioned conceptualization of UI may lead to years of agonizing accommodation and gradual tolerance of UI symptoms. In self-managing their UI, older patients place themselves at heightened risk for other medical complications by restricting fluid intake and reducing medication adherence, especially to diuretics. Because of these reasons, there is a need to overcome barriers to UI treatment. Even though increased media attention to UI and greater exposure to primary care services have helped generate opportunity for sufferers of UI to seek treatment, the published evidence suggests that many people are still not making use of such opportunities. These trends are exemplified by research suggesting that increased exposure to health professionals does not necessarily translate into increased reporting of UI symptoms [19]. Due to the unique and unconstructive nature of perceptions regarding UI among the general population, it is important for health care providers to be able to appropriately screen for UI when given the opportunity.

#### 1.2.2. Existing Tools to Measure UI

Since UI is multidimensional in scope, many self-report measures have focused on capturing the facets constituting UI in lieu of utilizing basic principles of survey construction and methodology for use in assessing older populations. Assessing UI in older women requires mindfulness of the schism that exists between the medical community and the populations whom they serve with regard to attitudes and beliefs about UI [20]. This consideration extends to the doctor-patient relationship as older adults may fail to report involuntary urine loss because they believe that it is not a legitimate medical concern or perceive reporting UI as a threat to their self-esteem [17]. During UI assessments, respondents' attitudes and feelings can impede motivation to address incontinence issues. When motivation is of concern, interviewer feedback can serve to legitimize the importance of reporting UI while encouraging recollection, even if the material is perceived as embarrassing [21]. The following is a summary of the most commonly used tools to assess UI. 

*Urogenital Distress Inventory and Incontinence Impact Questionnaire*. Expanding from the prior work of Norton [22], Shumaker et al. [23] developed the 19-item Urogenital Distress Inventory (UDI) in conjunction with the 30-item Incontinence Impact Questionnaire (IIQ). As some of the most highly cited tools within the UI literature, the UDI and the IIQ were developed in an attempt to capture both urological distress and UI-specific health-related quality of life (HRQOL). The 19 items of the UDI reflect 19 symptoms associated with lower urinary tract dysfunction. This self-administered measure asks respondents to assess the degree to which each itemized symptom “bothers” them on a 4-point Likert scale ranging from 1 = “not at all” to 4 = “greatly.” Using Factor Analytic techniques, these items have been grouped into one of three subscales: Irritative Symptoms (9 items), Obstructive/Discomfort (11 items), and Stress Symptoms (2 items). The IIQ consists of 30 items; 24 items are activity-based; thus they are intended to elucidate the negative impact that UI has on the respondents' engagement in a range of activities (e.g., entertainment, shopping, and recreation). The remaining 6 items of the IIQ are intended to identify UI's impact on various feelings (e.g., fear, frustration, and anger). Like the UDI, the IIQ utilizes a 4-point Likert format ranging from 1 = “not at all” to 4 = “greatly.” Using the same statistical methods as above, the authors generated four subscales for the IIQ: Physical Activity (6 items), Travel (6 items), Social Relationships (10 items), and Emotional Health (8 items). The above-mentioned two tools are described by their authors as being “psychometrically strong” and better at discriminating among UI patient groups than generic HRQOL measures [23].

There are several shortcomings of the UDI and IIQ, with the most glaring of these being the makeup of the sample population on which development and validation of the UDI and IIQ were conducted. To specify, the sample population consisted of 162 community-dwelling women recruited in the Southeast USA to participate in one of three clinical studies. Although care was taken to ensure that these women were qualified for a diagnosis of UI (i.e., via comprehensive clinical assessment, urine culture, cystometry, urodynamic evaluation, standardized pad testing, and the completion of a one-week UI diary), descriptive data reveals a highly homogeneous sample, with 95.7 percent of the research participants being white, 78.4 percent having been educated beyond high school, 62.3 percent married, and—of the 95.6 percent who reported their income—31 percent made in excess of 75,000 USA dollars per year. The characteristics of this sample make it very difficult to generalize the research findings in question to community-dwelling older women, who typically have much lower SES.

Reliability was assessed for both the UDI and IIQ using Cronbach's alpha coefficients. These values ranged within the “fair to good” range, with one subscale of the UDI, “Stress Symptoms,” demonstrating poor reliability (0.48). The UDI subscales' reliabilities (in parentheses) were Stress Symptoms (0.48), Irritative Symptoms (0.70), and Obstructive Discomfort (0.77). For the IIQ, reliabilities were Emotional (0.90), Physical Activity (0.87), Social (0.90), and Travel (0.87). In an attempt to rectify statistical shortcomings, van der Vaart et al. [24] analyzed the scale construction and validity of the UDI and IIQ on a random sample of *n* = 2,042 women along with a clinical sample of *n* = 196 women. Despite the fact that the vastly increased sample size helped alleviate concerns regarding the use of factor analytic techniques employed by Shumaker et al. [23] on small sample sizes (i.e., rule of 500; Comrey and Lee [25], with 100 = poor, 200 = fair, 300 = good, 500 = very good, 1,000 or more = excellent), the sample was again extremely homogonous in terms of ethnicity and was all Dutch speaking. The sample gathered by van der Vaart and colleagues was 98 percent white for the nonclinical sample and 97 percent white for the clinical one, with similar education and marriage demographics as those used in Shumaker et al.'s study [23]. Although the authors were able to demonstrate good internal consistency and reliability values, they had to add some new subscales based on the results of the factor analyses, perhaps due to having translated the scales into Dutch. The authors did, however, criticize Shumaker and colleagues for using urinary tract infection and urinary obstruction as exclusion criteria, stating “Excluding these women while developing a questionnaire on urogenital symptoms somehow seems odd” (pp. 102). Thus, the inclusion of women suffering from a wide array of urogenital conditions might have led to the development of different subscales.

In 2008, Sheward et al. [26] tested the two tools on a Scottish population: different short forms emerged, as the author developed subscales for the UDI and IIQ with greater stability, with the resulting 11-item UDI and 11-item IIQ models showing good internal consistency. Short forms of the UDI and IIQ were also generated by Uebersax et al. [27] who reduced the UDI from 19 items to 6 items (UDI-6) and the IIQ from 30 items to 7 items (IIQ-7). These short forms were intended to reduce respondent burden, cost, and assessment time. Using the original long forms, the authors utilized multiple regression analyses to find the items that best predicted the overall subscale score. They utilized data from the original *n* = 162 women who were used in the development of the long forms in Shumaker et al.'s study [23]. In order to assess the validity of these short form questionnaires, correlations were employed to determine how closely related the short form items were to the original long form subscales. The IIQ-7 total score correlated .97 with the IIQ total score. IIQ-7 items taken from specific subscales of the IIQ were also correlated with their respective IIQ subscale origins and demonstrated correlations ranging from .88 to .94. The UDI-6 total score correlated .93 with the UDI total score. UDI-6 items taken from specific subscales of the UDI were also correlated with their respective UDI subscales of origin. UDI-6 item correlations were .86 and .84 with the Irritative Symptoms and Obstructive/Discomfort UDI subscales, respectively. Correlation between the UDI-6 and the UDI Stress subscale was 1.0 as they were the exact same items. Unfortunately for the statistically adequate development of the UDI-6 and IIQ-7, the same homogenous sample of women used to validate the long forms was used to validate the short forms. Finally, the use of correlations between the short and long forms still does not tell us how these short forms would perform in a clinical practice setting, since they were never actually administered to anyone.

*Kings Health Questionnaire*. The Kings Health Questionnaire (KHQ) [28] is a condition-specific 21-item instrument that reflects a shift in clinical assessment for UI by acknowledging the discrepancies between patients self-reports and urodynamic tests. This notion holds that a woman's perceptions of her symptoms do not necessarily reflect actual symptom severity as measured by pads or other urodynamic test. The KHQ is comprised of seven domain-specific subcategories (i.e., emotional problems, role limitations, severity measures, social limitations, personal limitations, sleep/energy disturbances, and physical limitations). Even though the KHQ addresses the issue of perception as well as severity, aspects of the methodology employed for sample recruitment make interpretations of this instrument's efficacy for UI screening somewhat difficult to assess. The participants, 293 women referred for urodynamic testing at a tertiary uro-gynecological clinic in London, were asked to complete the questionnaire at home. The mean age of participants was listed as 51.4 years with no other demographic information reported. Internal consistency of the KHQ was strong, with subscale alphas ranging between (.72) and (.89). The authors suggested that this instrument is easy to complete and understand, based on the 97.3% completion rate; thus, this tool is well-suited for use in urodynamic clinics. It is noted, however, that this high completion rate could also be the result of the sample being comprised of women attending a clinic for urodynamic assessment. Furthermore, researchers noted that the 21-item length could be problematic with regard to both time and completion rates [29, 30]. 

*The Incontinence Severity Index.* The Incontinence Severity Index (ISI), developed by Sandvik et al. [31], represents the first empirical attempt to quantify UI severity. These authors intended to create a tool that covered only UI frequency and quantity. Thus, the ISI is comprised of just two items: (1) how often do you experience urinary leakage? and (2) how much urine do you lose each time? The first question is on a 4-point Likert type scale ranging from “less than once a month” to “every day and/or night.” Quantity is assessed via item two, a dichotomous item that allows participants to choose between “drops or little” and “more.” The severity index is created by multiplying the results of questions 1 and 2 scored as follows: 1-2 indicates slight UI, 3-4 moderate, and 6–8 severe UI. This index was validated with urodynamic pad tests, a method hypothesized to produce better agreement than had been noted in prior attempts at validation of anamnestic criterion of UI severity. The authors obtained significant correlations for both reported frequency (*r* = .32, *P* < .001) and reported amount of leakage (*r* = .37, *P* < .001) with pad weigh tests. There was, however, considerable overlap between those two categories, and the ISI was not subjected to psychometric testing. Furthermore, most researchers have pointed to the inherent difficulties that arise when trying to measure quantity through self-report measures. In 2000, the aforementioned authors modified the ISI slightly by changing item 2 from being dichotomous to a 3-point Likert type scale format, thus adding one more severity category, that is, “very severe.” Another problem is the lack of a 0 point (which we have in our UI screener tested herein), that is, lack of incontinence symptoms, as question 1 does not have a choice for the experience of no incontinence. Thus, it is possible that nonincontinent individuals, although without UI symptoms, could check the choice corresponding to experiencing a UI accident less than once per month. Thus, unless this tool is used on patients with incontinence symptoms, there is no way of knowing whether a woman has no such symptoms, and its use could potentially overestimate UI prevalence.

Overall, the previous summary of the available psychometric literature on UI measures has revealed several problems with the currently available choice of UI instruments; for instance, as already mentioned, the last measure described, although very brief and thus ideal for use in high-paced medical settings, was never tested psychometrically. In an attempt to fill some of the gaps in the current UI literature, in the present study we planned on testing in aim (2) the psychometric properties of our new UI screener on a nonclinical and ethnically diverse sample of older women, thus contributing substantially to the psychometric literature on UI screeners.

#### 1.2.3. Depression

Of the various conditions that have been repeatedly identified in the literature as being comorbid with UI, the most notable psychiatric condition continues to be depression [32, 33]. Evidence of the strong relationship between depression and UI is not limited to cases in the USA, as the universality of this phenomenon appears to be evident, given findings from a variety of other nations including the developing world [32, 34]. Explanations proposed to account for the etiology of these two debilitating disorders functioning in concert have taken a variety of forms, from psychiatric suggestions of UI being a psychosomatic manifestation [35] to more established linkages suggesting that the stigma, emotional distress, and loss of control associated with UI predispose the individual to becoming depressed [33, 36]. The latter of these hypotheses is strengthened by findings suggesting that younger women between the ages of 18–44 living with UI are at a threefold risk of being diagnosed with major depression in comparison with women over the age of 45 [7].

Whereas psychiatry-based theories for the development of the comorbidity in question seem reasonable, a more innovative approach suggests that the neurochemical dysfunctions associated with depression may, in some way, also contribute to issues related to micturition [33]. There is a wealth of empirical evidence suggesting an association between the neurological processes responsible for both mood symptomatology and micturition. Some of the most compelling evidence has come from recent developments in pharmacological treatments for UI. In the past, drugs designed to treat UI were regarded as having poor efficacy while exposing patients to side effects. These first generation antimuscarinics worked as anticholinergic drugs that inhibited the effects of acetylcholine on acetylcholine receptors via competitive inhibition. Two of the most pressing concerns regarding the efficacy of antimuscarinics involve bioavailability and side effects [37]. Tertiary amines like atropine are well absorbed and pass readily into the central nervous system (CNS). However, this high bioavailability lends to increased risk of side effects such as blurred vision, nausea, paralytic ileus (intestinal paralysis), and mental confusion, to name just a few [38]. These side effects can be profound in older age even after taking very small doses. On the other end of the spectrum, quaternary ammonium compounds such as glycopyrrolate exhibit a decreased incidence of CNS side effects (but not necessarily peripheral side effects). Yet, they are less readily absorbed and thus may lack the bioavailability necessary for the effective treatment of UI [39]. Given the side effects of anticholinergic drugs, certain populations are not ideal candidates for treatment with these drugs, with children and older adults being perhaps the most vulnerable to the adverse effects of anticholinergic drugs.

The successful use of tricyclic antidepressants (TCA) in treating UI has led researchers to reexplore the treatment of micturition deficiencies [40]. TCAs are well established for use in treating major depression due to their inhibition of the presynaptic reuptake of serotonin (5HT) and norepinephrine (NE) according to Karch [38]. The most widely recognized TCA for the treatment of UI is imipramine, which has been successfully employed in treating nocturnal enuresis in children along with stress UI [41]. Researchers conducting animal investigations into the mechanisms underlying TCA efficacy in treating UI have focused on the anticholinergic properties of imipramine; yet, the exact mechanisms underlying its efficacy are still poorly understood [37, 41]. There is, however, considerable reason to believe that imipramine's effect on 5HT may also play a significant role. Given that 5HT is known to produce an inhibitive effect on the commencement of micturition [40] by stimulating 5HT_2_ receptors, which increase striated sphincter closure [42], it is difficult to tease apart the multifaceted modes of action in which imipramine elicits its effect on UI. What is known, however, is that there has been a lack of consistent randomized controlled studies on imipramine; as a result, at a recent International Consultation on Incontinence, imipramine was given a “D” recommendation grading due to the need for more empirical research [41].

Over the past decade, there has been a surge in both animal and human studies on the roles of 5HT and NE in the neural control of micturition. Animal research involving cats has demonstrated that 5HT can influence somatic bladder activity by inhibiting parasympathetic activity [41]. Research on both cats and rats has established that the facilitation of bladder activity could be initiated by administering the serotonergic neurotoxin, 5,7-dihydroxytryptamine (5,7-DHT) [43, 44]. The most current and promising evidence on this topic is the recent advent of Serotonin Norepinephrine Reuptake Inhibitors (SNRI) having success in the treatment of certain forms of UI. Among the SNRIs, one drug in particular has been the primary focus of UI research, the drug Duloxetine [22]. Duloxetine has also shown a pronounced efficacy in treating depression, generalized anxiety disorder, neuropathic pain, diabetic neuropathy, and fibromyalgia [45]. It is believed to exert its influence on micturition by generating synaptic accumulation of 5HT and NE within Onuf's nucleus located in the sacral region of the spine, a region rich in motor neurons along with being the origin point of the pudendal nerve which innervates the genitalia along with the bladder and rectum [41]. Stage III clinical trials aimed at evaluating the efficacy of Duloxetine in treating UI have demonstrated a highly significant decrease in the episodic frequency of incontinence [46] along with a significant improvement in symptoms among women suffering from mixed UI [47]. The apparent effectiveness of Duloxetine in the treatment of major depressive disorder, neuropathic pain, and lower urinary tract disorders has led some to conclude that these distinct pathologies share a common pathophysiological basis [42].

Back in 1925, physiologist Barrington [48] published “The effect of lesions of the hind- and mid-brain on micturition in the cat.” This paper was the first to implicate specific brain regions as being critical components to voiding [49]. Recent advents in imaging technologies have allowed researchers to move beyond lesion studies and actually measure changes within regions identified as crucial to the micturition process. Within the mid-brain, an area known as periaqueductal gray (PAG) is considered to be the integrative brain center that relays ascending bladder information to a variety of other brain areas including the pontine micturition center (PMC), also known as Barrington's nucleus. Located in the pons, the PMC relays descending projections to the parasympathetic nucleus within the spinal cord [50]. The use of Functional Magnetic Resonance Imaging (FMRI) has confirmed that the PAG and PMC play key roles in micturition and continence [51]. There are still some questions as to the precise workings of the PAG and PMC; many of these questions have been raised due to the majority of this research being animal research [52]. Also, findings from human case studies where damage to the PAG has resulted in effects on continence conflict with what has been observed in rats and cats [53].

Along with the PAG and PMC, a variety of other brain regions have been identified as influential to the micturition process. Advanced imaging studies have identified the medial prefrontal cortex, cerebellum, and basal ganglia as being involved in the control of micturition, while the parietal cortex and limbic system have been implicated in the inhibitory voiding control mechanism [50, 51]. Some children who have undergone a medulloblastoma (removal of a tumor in the cerebellum) suffer from urinary incontinence; this indicates that cerebellar dysfunction also affects bladder function [51]. Although the PAG serves as the neural hub controlling micturition, it receives dense afferent projections from regions such as the amygdala and hypothalamus [54]. These regions, many of which are part of the limbic system, have all been identified as playing a major role in our emotions [51, 54–56]. When one observes how micturition serves to assist in certain sociosurvival behaviors such as relaying sexual and territorial information among various animal species [54], the neural interdependence between the brains micturition and emotional centers begins to seem logical from an evolutionary perspective. In 2009, Apostolidis and Fowler [50] devised a schematic illustration of the theoretical integration between the PAG, PMC, and a variety of higher brain regions. The authors acknowledged that the direction of the relationships that are not identified is unknown. Another dilemma facing researchers is that our knowledge regarding higher brain areas in micturition is relatively new and still incomplete [51]. Even though it is clear that the neurochemicals and neural regions essential in the function of emotion are also involved in micturition, we must be careful to recognize that most of the data that we have to date implicating a biological relationship between depression and UI is correlational.

#### 1.2.4. Existing Models of the Relation between UI and Psychopathology

Multiple models have been proposed to illustrate the relationship between UI and psychopathology. Thor et al. [42], in 2007, proposed a theoretical relationship between depression, pain, micturition, and the neurotransmitters 5-HT and NE. Depression is typified by a dysregulation of 5-HT and NE within the brain. Pain perception can be impacted by 5-HT and NE, which modulate pain sensitivity within descending pathways. Finally, lower urinary tract function is controlled by the CNS by way of neurotransmitters. Again, 5-HT and NE exert their influence within the lower urinary tract by influencing closure of the urethral sphincter. Although the relationships between these factors are conceptualized as correlational rather than causal, this illustration helps demonstrate the interdependence that these psychological and somatic processes share within the CNS. Of note is the importance of 5HT and NE in regulating a variety of functions along the CNS. So far, researchers have focused on Caudal CNS functions. The brain, however, plays a major role in both the physiological and psychological aspects of micturition. Recent advents in imaging technologies have allowed scholars to examine the brain regions believed to be crucial in the control of the micturition reflex.

If there is a biological basis for the comorbidity of UI and depression, it would most probably not exist in a vacuum. Few morbid conditions are singular in nature; rather, multiple interacting factors tend to contribute to deleterious health conditions [57]. In the case of UI, a “vicious circle” could ensue where emotional, behavioral, cognitive, and social aspects of incontinence begin to play on each other in a pathological fashion [36]. This circular pattern could then interact with innate functions to exacerbate interconnected neurobiological systems. This model, purposed by Perry et al. [36], demonstrates how real life cognitions and behaviors could help fuel the depression/UI phenomenon. In this model, cognitive behavioral factors such as internal and external triggers (e.g., bladder filling/sound of running water) can produce a perceived threat of accidentally wetting oneself if a toilet cannot be reached in time. This source of anxiety would work in a cyclical fashion by causing the individual to selectively attend to bodily events involved in micturition and producing cognitive misinterpretations of these somatic symptoms such as the belief that one has a weak bladder. These misinterpretations and somatization are further aggravated by avoidance and safety behaviors such as frequent bathroom trips, limiting fluid intake, and avoiding new places where a toilet may be inaccessible. Finally, the “vicious circle” just described is further aggravated by a symbiotic relationship between emotional consequences of UI (e.g., an increase in depression, shame, and anxiety, with a drop in self-confidence and self-image), social consequences of UI (e.g., isolation, stigma, and the threat of institutionalization while limiting daily routines and interpersonal interactions), and the consequences posed by a failure to seek treatment or a failure of appropriate care by case treatment (e.g., management versus treatment and exploring all treatment options). This model does well in thoughtfully disassembling the likely multifaceted etiology of UI.

### 1.3. Hypotheses and Research Questions of the Present Study

The hypotheses and research questions of this study were as follows: for aim (1), we intended to calculate the prevalence of UI in community-dwelling, ethnically diverse older women. Given that in the available literature on UI we did not find any studies on nonclinical populations of older women, we did not formulate a specific hypothesis for this aim. The lack of attention afforded to (a) ethnic minorities residing within the United States as well as (b) community-dwelling older women lends credence to the necessity of prevalence estimates for these populations. Concerning aim (2), we intended to test the reliability of our new UI short measure and expected our tool to be a reliable incontinence screener. We hoped to achieve an internal consistency finding of at least .70, which would have meant that this measure is highly reliable as stated by Tabachnick and Fidell [58]. The empirical evidence on UI assessment briefly summarized above demonstrates the need for UI instruments to be tested on heterogeneous populations, and especially on older women from various ethnic backgrounds who are living independently. Judging from the methodologies employed in the available UI assessment studies, our UI screener is the first one to be tested for reliability in a study on a community-dwelling sample of multiethnic older women in which urogenital dysfunction was not used as a prerequisite for inclusion. Although many of the authors of the instruments presented within our literature review claimed that their measures could be used for both identification and assessment of UI, validation of these tools on disease-specific populations makes it difficult to be sure that use of these instruments would indeed allow researchers and clinicians to identify UI among populations in which the presence of urogenital dysfunctions is unknown. Lastly, the third aim of this investigation was to ascertain whether there was a significant relationship between UI and depression in our sample, while controlling for age. This finding was expected even though relevant prior literature on this topic targeted primarily Caucasian clinical samples, while our sample is nonclinical and highly multiethnic. Observed relationships between UI and depression among nonclinical populations are necessary to support generalizability of such comorbidities beyond the clinical setting. Furthermore, the potential impact that culture may have on empirical observations of the UI-depression link within multiethnic populations lends support to the salience of the present investigation.

## 2. Methods

### 2.1. Participants

In a 4-year study funded by NIH from 2006 to 2010, noninstitutionalized older women residing in Los Angeles County were recruited using care to ensure that participants did not reside in an assisted care facility. We did this in order to collect a sample that was cognitively high functioning, thus more representative of older women who are able to live in the community independently. In order to obtain a diverse sample, both ethnically and with regard to social participation, a purposive sampling method was utilized. This practice helped generate a sample that was highly representative of community-dwelling older women. Recruitment of participants was conducted by directly approaching older women at churches, stores, and libraries.

Data collection took approximately three years, and the careful recruitment efforts were intended to ensure a sample that included women who were socially isolated, thus not likely to take part in research endeavors. Recruitment was limited by the following criteria: (a) minimum age of 60; (b) fluent in speaking and understanding English; (c) community-dwelling (noninstitutionalized); and (d) able to provide informed consent.

### 2.2. Procedure

To ensure that participants were comfortable, care was taken to ensure flexibility when determining where and when interviews took place. Interviewers met with participants at their homes and at community locations like senior centers and libraries according to the preference of each participant. Typically, each assessment session lasted over one hour, and care was taken to reduce respondent burden by having interviewers read aloud each question and record respondent's answers by hand. Older women were also given a break mid-way through the interview to minimize fatigue.

In order to offset disclosure concerns and mistrust, both gender and ethnicity were taken into account, in that we matched interviewers to participants as suggested by Ballard et al. [59]. Finally, interviewers were trained by the first author in active listening techniques along with interviewing skills and cultural competence. This study met approval by the Institutional Review Board of California State University, Northridge.

### 2.3. Assessment Tools

#### 2.3.1. Cognitive Screener

In order to screen out major cognitive dysfunction and dementia, we used the Mini-Cog [60]. Demonstrated to be a valid tool for use with older ethnically diverse populations [61], this short tool allows for the rating of participants based on their completion of simple memory tasks and a clock drawing component with assigned scores of 2 if “normal” or 0 if “abnormal.” Concerning the clock part of the tool, a normal/2 score requires participants to draw the time 11:10 with correct order and direction of numbers, along with correct spacing of numbers so that they fit within the clocks borders. With regard to the second component of the measure, the test requires the recall of a set of words; successful recall is scored from a range of 0 to 5. A total score of 3 or above indicates no major impairments of cognitive ability. This tool was only used for the purpose of screening for major cognitive dysfunction in our sample (in order to exclude those with such problems); thus there is no data to report on respondents' answers to the Mini-Cog assessment. All the women approached achieved a score of 3 or higher.

#### 2.3.2. Demographics List

A brief yet comprehensive demographic self-report list developed by the first author contained 10 basic-information items. The items, which generated nominal, ordinal, and interval data, inquire into the participants' ethnicity, age, education, income, years of residence within the United States, employment, and marital status. Completion of the demographic list takes approximately five minutes and was conducted by paper and pencil. This brief demographic tool has been used extensively by the first author in behavioral medicine research at California State University, Northridge.

#### 2.3.3. The “Center for Epidemiological Studies-Depression Scale” (CES-D)

With the ability to differentiate between multiple facets of mood functioning, the CES-D is a 20-item self-report tool containing 4 subscales: (1) depressed affect, (2) positive affect, (3) interpersonal distress, and (4) somatic/vegetative signs. The CES-D has demonstrated high internal consistency among both clinical and nonclinical populations of men and women across various ethnicities and has also been used to study geriatric populations [62, 63]. With a Cronbach's alpha coefficient of .85, Spearman-Brown of .88, split-half reliability at approximately .79, and good test-retest reliability [64–66], this instrument was designed to assess depressive symptomatology within the context of more immediate life circumstances. Participants are asked to rate the frequency with which they experienced the specified symptoms in the past week: 0 = rarely or none of the time; 1 = some or a little of the time; 2 = occasionally or a moderate amount of time; 3 = most or all of the time. Scoring of the CES-D consists of adding the total from each scale together such that a score between 16 and 22 suggests mild depressive symptomatology, and scores exceeding 23 suggest significant depressive symptoms.

#### 2.3.4. A New UI Screener for Older Adults

This is an original tool created by the first author as a needed brief screener of UI symptomatology to be used in fast-paced medical and psychological care settings. It is comprised of three questions, which are (1) approximately how many incontinence problems do you experience a month?, (2) I find my incontinence to be emotionally stressful, and (3) I believe that my incontinence limits my social activities. Items 2 and 3 ask respondents to assess the degree to which each itemized symptom impacted them on a 4-point Likert-type scale, with 0 = not at all, 1 = almost not at all, 2 = a little, 3 = moderately, 4 = significantly, and 5 = extremely.

The tool tested herein, although very brief, has a methodologically sound foundation in view of the following rationale. Utilizing focus groups, Fultz and Herzog [17] developed a series of suggestions for the development and employment of surveys addressing UI. One of the things that they considered was the assessment of UI severity through survey interview. According to the above-mentioned authors, severity of UI consists of three factors: psychosocial impact, frequency of urine loss, and quantity of urine loss. Quantity, which refers to actual volume loss, can be very difficult to ascertain through self-report methods. Respondents presented with questions regarding metric volume loss have difficulty estimating such quantities because they are unlikely to have ever urinated into a measuring device. Frequency, however, can be a more reliable assessment of severity when conceptualized within an appropriate time frame. Via item 1 of our screener, respondents were asked “approximately how many incontinence problems do you experience per month?” This time frame has been reported as ideal when requesting episodic frequency of UI among older populations [17]. Quantification of this item was handled by assigning a response of 0 to “not at all,” once a month to “almost not at all,” two times a month to “a little,” three times a month to “moderately,” four times a month to “significantly,” and five times a month or more to “extremely.” Due to the overwhelming concern regarding the uniformity of severity categorization noted in the literature, the general rule of one or more incontinence accidents per week was adopted as the cutoff for significant and extreme [31, 67, 68].

The final factor of significance when evaluating UI severity is psychosocial impact. Although the International Continence Society definition of incontinence as an “involuntary loss of urine that is objectively demonstrable and a social or hygienic problem” [69] has changed to exclude the objective demonstration of social and hygienic problems, most researchers concur that a patient's psychosocial health is an important aspect of the severity of UI [70]. Our screener assesses social limitations with the item “I believe that my incontinence limits my social activities.” This item alone, however, may not be enough to evaluate the psychosocial impact of UI, because individuals who have learned to self-manage their UI through accommodation may no longer be limited in their social activities, yet they still feel emotionally distressed by their condition. This dilemma is addressed by use of the screener's item “I find my incontinence to be emotionally stressful.” In theory, this would allow for the identification of individuals who may not self-identify as “incontinent” due to their ability to self-manage accidents (while they still experience psychological distress).

In view of the above-mentioned issues, our new screener had potential to be a solid UI short measure. Furthermore, findings of prior research on how the presence of an interviewer can significantly increase reporting of health concerns [21] lend support to the administration of a screener of this kind in the presence of a trained interviewer, as done in the present study. Moreover, we instructed research assistants who conducted the clinical assessments to explain that urinary incontinence is “unwanted urination” and to “provide examples if necessary.” This instruction within the protocol helped ensure that the respondent understood what was being asked.

## 3. Results

In the analyses for aims (1) and (3), the original sample size of *N* = 140 was reduced to 131 with the deletion of seven cases with significant missing data and two cases that did not qualify as either continent or incontinent (answered “N/A”). A missing value analysis was performed to impute two missing scores on item 3 of the CES-D for two participants. One age value was imputed using missing values analysis for the covariate age. In order to properly assess CES-D scores, the “positive affect” subscale was reverse-coded in a new variable as directed by Radloff and Teri [64]. Concerning the sample size for aim (2) only, we used a smaller sample of *N* = 119 participants with complete data. Frequency analyses on the demographic characteristics of the sample used in the MANCOVA analyses (*N* = 131) revealed a highly heterogeneous sample, as illustrated in Table 1. The ethnic makeup of the women in our sample consisted of 10.7% who were identified as African American, 16.0% as Asian American, 29.1% as Hispanic (17.6% as Mexican American and 11.5% as other Hispanic/Latinas), and 44.3% as European American. Income levels among participants were also very diverse, with 28.2% of the respondents reporting a total household income below $20,000 annually.

Aim (1) frequency data analyses revealed that 28.2% of older women in our sample experienced UI symptoms. Concerning aim (2), a reliability analysis for the three UI items utilizing Cronbach's alpha was performed. This analysis revealed a Cronbach's alpha of .75, which is more than acceptable [71], especially given the concise nature of our screener. Moreover, the internal consistency statistics reported in Table 2 indicate that the removal of any of the three UI items would have not produced any major improvements to the overall internal consistency of .75. Removal of item 3 would yield a very small positive impact on internal consistency (alpha 0.78), but given the potential psychosocial significance of this item, its removal is not needed.  Table 3 displays the interitem correlation matrix with all the correlations between UI items, as well as the item-total correlations. Statistically significant correlations were found between all UI items. Specifically, correlations were significant between the total obtained on the UI screener and item 1 (*r* = .688, *P* ≤ .01), item 2 (*r* = .689, *P* ≤ .01), and item 3 (*r* = .611, *P* ≤ .01).

To ascertain whether there was a significant relationship between UI and depression in a nonclinical multiethnic sample of older women, we planned on conducting a two-group between-subjects multivariate analysis of covariance (MANCOVA) comparing four dependent variables corresponding to the four subscales of the CES-D, that is, depressed affect, positive affect, somatic/vegetative signs, and interpersonal distress along two independent variables of continent versus incontinent, while controlling for age. To prepare for these analyses, we first conducted a descriptive statistics exploration, which revealed moderate to substantial skewness among all four dependent variables as determined using *Z* tests (*Z* = (*S* − 0)/*S*_*s*_). SPSS also identified an extreme outlier in the somatic subscale and several extreme outliers in the interpersonal distress subscale. Outliers were readily alleviated using square root transformations. However, skewness on interpersonal distress was still significant; logarithmic and inverse transformations were still unable to correct the skewness on interpersonal distress. Therefore, it was decided that this variable, which was comprised of only two of the twenty CES-D items, would be excluded from the analyses. Our prediction relative to whether there was a significant relationship between UI and depression in a nonclinical multiethnic sample of older women was not supported, as illustrated by our MANCOVA results (displayed in Table 4) indicating a lack of significance.

In particular, the SPSS MANCOVA to ascertain whether there was a significant relationship between UI and depression in a nonclinical multiethnic sample of older women was conducted on the newly transformed DVs excluding interpersonal distress while controlling for age. *N* = 37 participants were identified as being incontinent, which accounts for 28.2 percent of the sample. Box's M was not significant, indicating that the assumption of covariance homogeneity across groups was met. Levine's tests of equality of error variances among DV's demonstrated that the assumption was met for somatization and positive affect variables but not for depressed affect [*F*(1,129) = 4.187, *P* = .043]. Use of Wilks' criterion demonstrated that there was no significant between-groups effect [*F*(3,126) = .439, *P* > .05]. The covariate age was also not a significant contributor to the overall model [*F*(3,126) = .178, *P* > .05].

To explore whether significant findings had not been achieved via implementing a MANCOVA due to the fact that (1) intercorrelations among depression components were all higher than .30 [i.e., interpersonal distress and positive affect (*r* = −.387, *P* < .01); somatic/vegetative signs and interpersonal distress (*r* = .637, *P* < .01); somatic/vegetative signs and depressed affect (*r* = .654, *P* < .01); somatic/vegetative signs and positive affect (*r* = −.458, *P* < .01); interpersonal distress and depressed affect (*r* = .491, *P* < .01); positive affect and depressed affect (*r* = −.521, *r* = .01)] and/or (2) we had not included interpersonal distress as a component of depression in the MANCOVA, we implemented post-hoc Roy-Bargmann step-down analyses, as recommended by Tabachnick and Fidell [58]. We used all four original totals from each subscale of the CES-D, including the total for interpersonal distress. Given the significant correlations between the four depression subscales, each of them was entered in order of theoretical importance, with higher priority depression subscales/DVs treated as covariates along with the covariates age. No significant relationships were demonstrated via the above-mentioned post-hoc analyses between incontinence and the CES-D subscales.

## 4. Discussion

To date, there has been a glaring underrepresentation of both nonclinical and multiethnic populations in UI investigations. In our study, less than half of our sample self-identified as European-American; thus, this is one of only a few available empirical investigations on the UI-depression link employing an ethnically diverse nonclinical sample. Also, many women in our sample had financial challenges, with approximately 3 out of 10 of them living with a total family income less than $20,000. In this regard, the USA poverty level for 2011 is $10,890 when living alone, $14,710 for a family of two, $18,530 for a family of three, and $22,350 (all total yearly income) for a family of four [72]. The makeup of our sample serves to address the paucity of available information pertaining to UI prevalence and to UI correlates (in this study's case, depression and demographic factors) in non-European-American populations.

**Figure 1:**
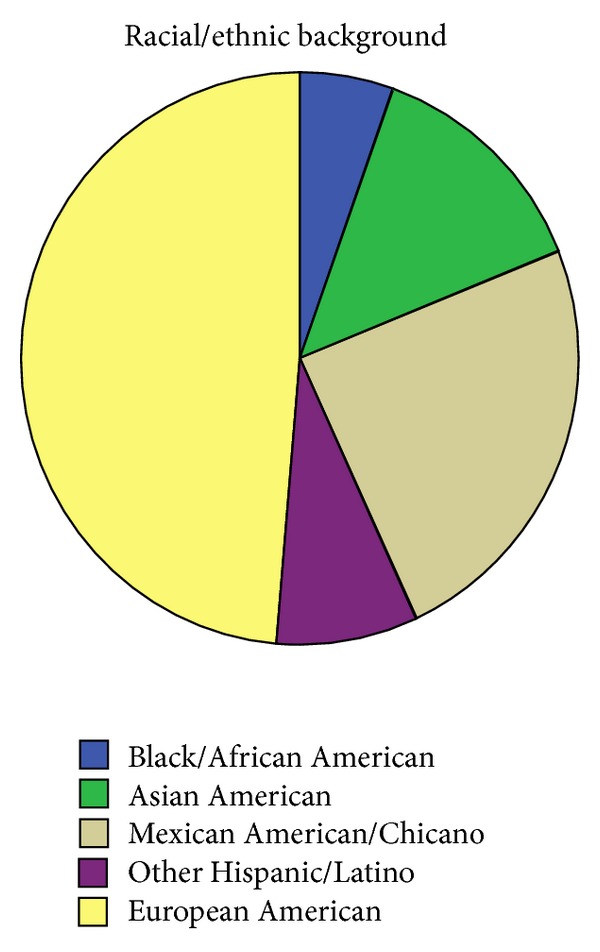
Ethnic composition of our sample.

In our first aim, we intended to establish the prevalence of UI given the inconsistencies observed in prevalence reports of UI worldwide. Data analyses revealed that 28.2% of our sample had UI symptoms. Of those self-identifying as incontinent, 51.4% were non-European-American. Hispanics (32.4%) comprised the greatest number of non-European-American incontinent cases. Furthermore, the prevalence of UI for the European-American population as a whole was 31%, while UI prevalence among all ethnic minority respondents was 26% (detailed information on the racial/ethnic groups represented in this study is reported in Table 1 and depicted in Figure 1).

Regarding the new UI screener tested in aim (2), development of UI-related instruments utilizing multiethnic community-dwelling populations has been greatly lacking [12–14]. This concern exists in lieu of evidence suggesting that culture can have a significant impact on a woman's perception of her own urinary symptoms [16]. The results of the analyses for this aim showed that this screener is a reliable measure for identifying UI symptomatology. It was particularly significant that the internal consistency coefficient was higher than .70, given that this coefficient is dependent upon the number of items in a measure and is typically higher when there are several items (typically more than three) in an instrument.

Results concerning aim (3) indicate that our hypothesis was not supported: incontinence did not have a significant relationship with depression as assessed by the CES-D in our sample. This is an intriguing result, given the characteristics of our sampling population; many speculations could be made regarding the reasons for this finding. First, despite the fact that biological explanations of the relationship between micturition and depression seem feasible, it is rare for morbid conditions to be singular in nature, given that several interrelated factors could contribute to negative health conditions [57]. This probably holds true for UI, as mediating factors such as culture or social support may have a strong influence on the relationship between UI and depression: for example, in cultures that view UI as natural and an expected part of aging, perhaps the psychological impact of UI could be smaller. Research is needed on this understudied topic.

Indeed, as the stigma associated with UI is challenged by greater awareness via both the media and clinicians' information of their patients, older women may have greater opportunities to address their UI symptoms. The latter possibility may indicate the increased need to control for the variable “currently receiving UI and/or antidepressant treatment” in future studies, as the treatment for UI is usually via antidepressants, and, by definition, this is the case for the treatment of depression. A literature review of this topic is well beyond the scope of this study, but one example of this empirical work is a study conducted by Zorn et al. [73]. These authors speculated that the significance of the depression-UI link obtained in their study could be due to altered serotonin function in patients with UI and could explain, at least partially, the efficacy of the use of serotonergic-based antidepressants in the treatment of at least one type of incontinence, that is, urge incontinence. If the latter conjecture is true, it could lend credence to the salience of fostering greater awareness of UI and depression treatment options for incontinent older women. Another potential factor that might have not allowed us to achieve significance for aim (3) is that we did not control for income (in part due to a modest sample size). Indeed, income may need to be controlled for, as it could be that older women with different levels of income could be more or less prone to depressive as well as UI symptomatology; research is certainly needed on this understudied topic.

The current study has many limitations beyond those mentioned above, such as the exclusive use of self-report data and the lack of medical data to corroborate reports of UI and/or depressive symptomatology. Moreover, we used a cross-sectional design; therefore, our findings do not imply causation. Furthermore, many potentially relevant variables were not assessed, such as medication use, acculturation, and disclosure to health providers of experiencing UI symptoms. Also, because medical treatment was not assessed in this somewhat preliminary study (to limit its scope), incontinent women in our sample might have been receiving treatment for their UI or their depressive conditions. This is a complex issue that might have affected our results, as being on a medication regimen might have had the side effect of reducing depressive symptoms and/or UI. Interested researchers should conduct longitudinal studies in this area, making sure to add the above-mentioned variables to their clinical assessment battery as well as other relevant variables to strengthen their research design, in order to shed additional light on the neglected topic of UI among nonclinical, ethnically diverse samples of older women.

## 5. Conclusive Comments

In the present investigation, we ascertained that a sizable percentage (28.2) of community-dwelling, ethnically diverse older women residing in Los Angeles County is living with UI symptoms. This is a clinical problem that needs to be addressed and further studied, as it has ramifications for the clinical practice of medical doctors and psychologists/clinicians dealing with older adult populations. More research is needed on whether UI is underdiagnosed and undertreated and, if this is indeed found to be the case, on reasons why it is occurring. Potential answers to this question include exploring cultural/ethnic issues and the related reluctance of many minority individuals to confide in health providers. It is possible that older women—from all ethnic backgrounds—feel too embarrassed about their UI symptoms to discuss them with a health provider and face living with UI without receiving treatment. On the other hand, cultural factors may play a role in shaping whether and how older women report and address UI symptoms. Our data analyses showed that the new UI tool proposed herein is a reliable UI screener. We recommend that interested scholars and clinicians consider testing the validity of this promising tool within community samples by using it in conjunction with other UI tools. Given that the UI-depression link was not significant in the present study, more research is needed to further elucidate the mechanisms linking depressive symptomatology and UI, especially when considering that prior research focusing on specific ethnic groups has shown that culture may affect how older women perceive UI.

## Figures and Tables

**Table 1:** Demographic characteristics of the sample (*n* = 131).

Ethnicity	
Black/African American	10.7
Asian American	16.0
Mexican American	17.6
Other Hispanic/Latino	11.5
European American	44.3

Income	
Less than $20,000	28.2
$20,000–$39,000	25.2
$40,000–$59,999	16.8
$60,000 or above	23.6
Refused to answer	6.1

Education	
Less than high school	20.6
Graduated high school	20.6
Trade school	7.6
Some college	25.1
Bachelor's degree	16.0
Post bachelors	8.4
Refused to answer	1.5

Age	
60–64	28.2
65–74	48.1
75–84	19.8
85+	3.8

Mean age = 69; minimum age = 60; maximum age = 90.

**Table 2:** Internal consistency statistics.

	Scale mean if item deleted	Scale variance if item deleted	Corrected item-total correlation	Cronbach's alpha if item deleted
Item 1	0.5546	2.639	0.652	0.633
Item 2	0.7647	3.198	0.713	0.508
Item 3	1.0504	5.455	0.532	0.786

Cronbach's alpha = 0.754.

**Table 3:** Interitem correlation matrix.

Item	Item content	1	2	3
1	Approximately how many incontinence problems do you experience a month?	—		
2	I find my incontinence to be emotionally stressful	0.658**	—	
3	I believe that my incontinence limits my social activities	0.439**	0.506**	—

	UI screener total	0.688**	0.689**	0.611**

**Correlation is significant at the 0.01 level (2-tailed).

**Table 4:** MANCOVA to verify aim (3).

Effect		Value	*F*	Hypothesis df	Error df	Sig.	Partial eta squared	Observed power
Intercept	Wilks' lambda	0.877	5.883	3.000	126.00	0.001	0.123	0.949
	Hotelling's trace	0.140	5.883	3.000	126.00	0.001	0.123	0.949
	Roy's largest root	0.140	5.883	3.000	126.00	0.001	0.123	0.949

Age	Pillai's trace	0.041	1.783	3.000	126.00	0.154	0.041	0.455
	Wilks' lambda	0.959	1.783	3.000	126.00	0.154	0.041	0.455
	Hotelling's trace	0.042	1.783	3.000	126.00	0.154	0.041	0.455
	Roy's largest root	0.042	1.783	3.000	126.00	0.154	0.041	0.455

UI	Pillai's trace	0.010	0.439	3.000	126.00	0.725	0.010	0.136
	Wilks' lambda	0.990	0.439	3.000	126.00	0.725	0.010	0.136
	Hotelling's trace	0.010	0.439	3.000	126.00	0.725	0.010	0.136
	Roy's largest root	0.010	0.439	3.000	126.00	0.725	0.010	0.136
